# Development of an Interdigitated Electrode-Based Disposable Enzyme Sensor Strip for Glycated Albumin Measurement

**DOI:** 10.3390/molecules26030734

**Published:** 2021-01-31

**Authors:** Mika Hatada, Noya Loew, Junko Okuda-Shimazaki, Mukund Khanwalker, Wakako Tsugawa, Ashok Mulchandani, Koji Sode

**Affiliations:** 1 Joint Department of Biomedical Engineering, The University of North Carolina at Chapel Hill and North Carolina State University, Chapel Hill, NC 27599, USA; mikah@email.unc.edu (M.H.); noya-loew@rs.tus.ac.jp (N.L.); jokudas@email.unc.edu (J.O.-S.); mukund@live.unc.edu (M.K.); 2 Department of Biotechnology and Life Science, Graduate School of Engineering, Tokyo University of Agriculture and Technology, 2-24-16 Naka-cho, Koganei, Tokyo 184-8588, Japan; tsugawa@cc.tuat.ac.jp; 3 Department of Chemical and Environmental Engineering, University of California, Riverside, CA 92521, USA; adani@engr.ucr.edu

**Keywords:** interdigitated electrode, glycated albumin, fructosyl amino acid oxidase, point of care testing, disposable enzyme sensor, diabetes mellitus

## Abstract

Glycated albumin (GA) is an important glycemic control marker for diabetes mellitus. This study aimed to develop a highly sensitive disposable enzyme sensor strip for GA measurement by using an interdigitated electrode (IDE) as an electrode platform. The superior characteristics of IDE were demonstrated using one microelectrode of the IDE pair as the working electrode (WE) and the other as the counter electrode, and by measuring ferrocyanide/ferricyanide redox couple. The oxidation current was immediately reached at the steady state when the oxidation potential was applied to the WE. Then, an IDE enzyme sensor strip for GA measurement was prepared. The measurement of fructosyl lysine, the protease digestion product of GA, exhibited a high, steady current immediately after potential application, revealing the highly reproducible measurement. The sensitivity (2.8 nA µM^−1^) and the limit of detection (1.2 µM) obtained with IDE enzyme sensor strip were superior compared with our previously reported sensor using screen printed electrode. Two GA samples, 15 or 30% GA, corresponding to healthy and diabetic levels, respectively, were measured after protease digestion with high resolution. This study demonstrated that the application of an IDE will realize the development of highly sensitive disposable-type amperometric enzyme sensors with high reproducibility.

## 1. Introduction

Glycated proteins, such as hemoglobin A1c (HbA1c) or glycated albumin (GA), are important glycemic control markers for diabetes mellitus. These proteins are the product of one of two possible nonenzymatic reactions: either a reaction between glucose and the N-terminal valine residue of hemoglobin’s β-chain or a reaction between glucose and lysine residues at the surface of human serum albumin. The level of HbA1c reflects the average blood glucose level over a period of 2–3 months, while the level of GA reflects the blood glucose level over a period of 2–3 weeks, depending on their life spans [1]. While HbA1c is currently the most widely used long-term glycemic control marker for diabetes, GA has advantages over HbA1c [2,3,4]. First, GA reflects the glycemic condition over a shorter period than HbA1c; thus, GA levels change rapidly according to the change in blood glucose level. Second, GA levels accurately reflect glycemic status under the condition of hematologic disorders such as anemia and variant hemoglobin, where abnormal HbA1c levels are observed. GA is expected to be utilized increasingly along with or as an alternative to HbA1c as a long-term glycemic control marker. Currently, in clinical applications, GA is measured with the enzymatic method at the central testing laboratory using the enzyme reagent for an autoanalyzer that has been commercialized by Asahi-Kasei Pharma (Tokyo, Japan) as Lucica^®^ GA-L [5]. In this enzymatic analytical kit, fructosyl amino acid oxidase (FAOx) is employed. First, GA is digested with protease, and released ε-fructosyl lysine (ε-FK) is oxidized by FAOx. Then, hydrogen peroxide produced through the enzymatic reaction is measured spectroscopically [6]. This enzyme-based GA measurement was first approved for the market mainly in Asian countries, such as Japan (from 2002), China (from 2003), Korea (from 2013), Indonesia (from 2013) and Taiwan (from 2015). Recently, enzyme-based GA measurement has also been approved in the EU (from 2015) and by the Food and Drug Administration (FDA) in the USA (from 2017). Therefore, the importance and usefulness of GA has been increasingly recognized worldwide. Although GA is a useful glycemic control marker, point-of-care testing (POCT) for GA has not yet been developed. Therefore, the development of a simple and rapid measurement method for GA suitable for POCT is required.

Electrochemical biosensors based on oxidoreductases have a wide application field in medical care and food and environmental protection. These biosensors are cost effective and enable rapid measurement. The most representative and commercially available disposable electrochemical enzyme sensors are those for the self-monitoring of blood glucose (SMBG), which are dedicated for the glycemic level control of diabetes mellitus. These biosensors are usually composed of enzymes, artificial electron acceptors (mediators) and disposable electrodes. SMBG is based on endpoint assays, where the sequential reaction of substrate oxidation with an enzyme and reduction of the mediator finish in several seconds, and the produced reduced-form mediator is measured with the chronoamperometry method. Previously, our group reported an SMBG-type endpoint assay-based disposable electrochemical enzyme sensor for the measurement of GA using FAOx, which is the same enzyme as used in the current enzymatic assay [7]. First, GA is digested by protease, and ε-FK is released. Then, FAOx oxidizes ε-FK, and the oxidized-form mediator is reduced simultaneously. The amount of produced reduced-form mediator is measured with the chronoamperometric method. As the electron mediator, hexaammineruthenium (III) chloride (a Ru complex) was used along with a disposable screen-printed carbon electrode (SPCE). To obtain the chronoamperometric signal, FAOx, the Ru complex and the sample were reacted on the electrode area for 1 min to oxidize the existing substrate (ε-FK) and produce a reduced-form mediator. Then, the potential to reoxidize the mediator was applied, and the current was monitored. The current response depending on the GA concentration was observed successfully; however, the sensitivity and period required for monitoring need to be improved. In the chronoamperometric measurement, the observed current response follows the Cottrell equation; thus, generally, the current decreases over time until the steady-state current is reached. The current values obtained at the fixed time, after the potential is applied (usually at several seconds), are used as the representative current values for each sample. Therefore, the sensitivity is crucially dependent on the current sampling time after the potential application. Additionally, there is the possibility of the time lag of the sampling time when different lots of electrochemical meters are used. Since the response current decreases with time, the sampling time lag caused by the meter resulted in poor reproducibility. Therefore, to achieve a higher sensitivity and a higher reproducibility of the measurement, the magnitude of the current values and the response time required to reach the steady state current should be considered.

Interdigitated electrodes (IDEs) are known as one of the geometries of microelectrodes and have received increasing attention. IDEs consist of two individual arrays of microelectrodes in an interdigitated configuration. These electrodes have been reported in amperometric biosensors employing dual potentiometry, where two IDEs of a pair are used as working electrodes (WEs), individually, to achieve highly sensitive sensors based on redox mediator recycling-based signal amplification [8,9,10,11,12,13]. For instance, one working electrode (WE1) is held at an oxidative potential, which drives the oxidation of the electrochemically active species, while the other working electrode (WE2) is held at a reductive potential to drive the opposite reaction, the reduction. Thus, species produced at one electrode diffuse to the other electrode, where they are converted back to their previous form. This process is called redox cycling and was first demonstrated by Bard et al. [14]. In this mode, a uniform concentration gradient of redox species between two WEs is formed immediately, and the reduced-form and oxidized-form species are continuously supplied to WE1 and WE2, respectively, by diffusion from each electrode. Therefore, a greatly amplified and steady current is obtained. The total amount of redox-coupled species can be measured with high sensitivity. In this dual mode, the collection efficiency, which is the ratio of current values obtained with WE2 to that of WE1, or in other words, the ratio of oxidized-form species that reach WE2 from WE1, is an important parameter when evaluating the signal amplification. This parameter is dependent on the distance between WE1 and WE2 [15]. Although dual potentiometry has often been applied for the detection of small quantities of redox species in amperometric sensors, dual potentiometry is not applicable to measure the concentration ratio of reduced-form and oxidized-form mediators in the mixture solution, which is the most common principle for disposable-type amperometric enzyme sensors employing electron mediators.

The superior characteristics of IDEs prompted us to use IDE as an alternative platform technology for disposable enzyme sensors, especially for SMBG-type amperometric sensor strips, without the employment of conventional dual potentiometry. The most prominent property of IDE is the distance (gap) between two individuals of microelectrodes in an interdigitated configuration; namely, the gap between the two electrodes is smaller (~50 µm) than the diffusion layer created during the redox reaction on the anode and on the cathode. Therefore, if each individual microelectrode of an IDE is used as a working electrode and as a counter electrode, the reaction at the counter electrode will keep the reduced mediator concentration on the working electrode almost constant. Thereby, a steady-state current will immediately be achieved, and a high electrical current will be observed in the sensor.

In this study, we aimed to develop a highly sensitive disposable enzyme sensor strip for GA measurement by using an IDE as the electrode platform. The superior characteristics of IDEs were investigated using one microelectrode of the IDE pair as the WE and the other as the counter electrode (CE) and by measuring different concentrations of ferrocyanide in a mixture with ferricyanide. The oxidation current immediately reached the steady state when the oxidation potential was applied to the WE. Then, an IDE enzyme sensor strip for GA measurement was prepared using FAOx and an Ru complex as an electron mediator. The IDE enzyme sensor strips showed a high steady current immediately after potential application, with a higher sensitivity than that of the SPCE-based enzyme sensor strip that we previously reported. This study demonstrated that the application of an IDE as an alternative electrode platform will realize the development of highly sensitive disposable amperometric enzyme sensors with high reproducibility.

## 2. Results and Discussion

### 2.1. Electrode Characterization

**Figure 1:**
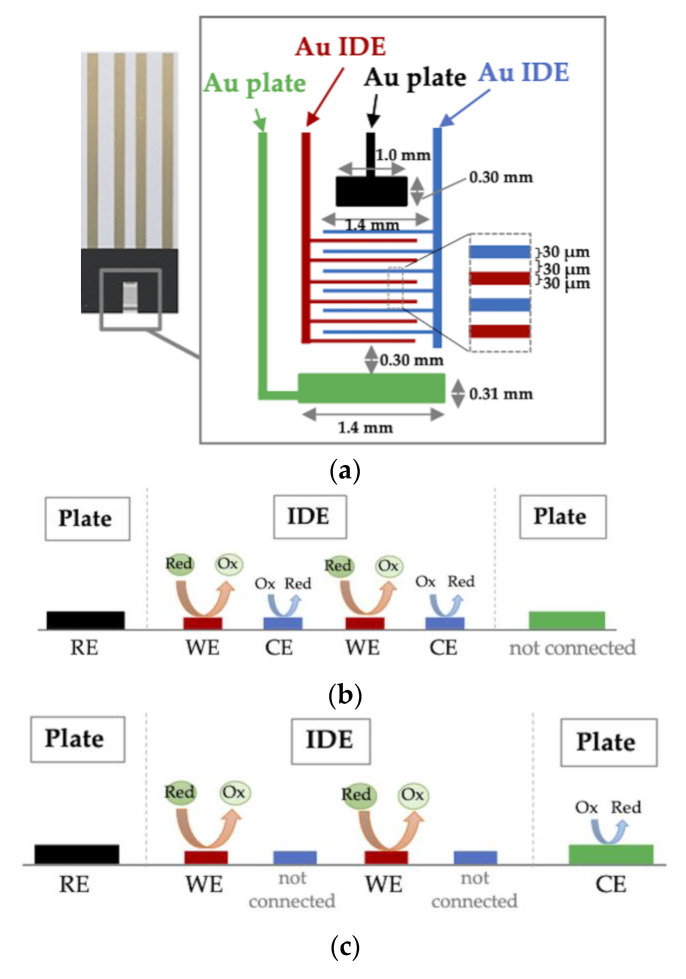
Configuration of the 4-electrode interdigitated electrodes (IDE) strip with the interdigitated array microelectrodes in the middle (red and blue), and two plate electrodes are at the top (black) and bottom (green) (**a**). Scheme of electrode assignments of IDE working electrode (WE)-IDE counter electrode (CE) mode (**b**) and IDE WE-plate CE mode (**c**).

**Figure 2:**
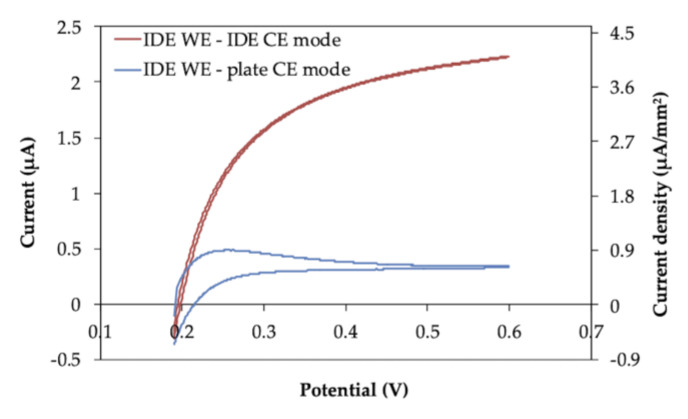
Cyclic voltammograms of IDE strip. Each cyclic voltammogram was obtained in the solution containing both 1 mM ferrocyanide and 9 mM ferricyanide, as a total concentration of 10 mM ferrocyanide/ferricyanide. Cyclic voltammogram in red was obtained using IDE with IDE WE-IDE CE mode, and the one in blue was obtained using IDE with IDE WE-plate CE mode, in 100 mM KCl. The sweep rate was 10 mV/s.

The superior characteristics of IDEs were first investigated by cyclic voltammetry (CV) measurement and chronoamperometry (CA) measurement of ferrocyanide in a mixture with ferricyanide. The IDE strip was configured in either IDE WE-IDE CE mode, where one microelectrode of the IDE pair was used as the WE and the other as the CE (Figure 1b), or IDE WE-plate CE mode, where one microelectrode of the IDE pair was used as the WE and the external plate electrode on the IDE strip was used as the CE (Figure 1c). The cyclic voltammograms are shown in Figure 2. As a result, the steady-state oxidation current without a peak, which was not diffusion limited, was observed in IDE WE-IDE CE mode (Figure 2, red line). In contrast, when one IDE was used as the WE and the plate electrode was used as the CE (IDE WE-plate CE mode), the peak current from oxidation of ferrocyanide, which was the diffusion limited current, was observed (Figure 2, blue line), and the current value was smaller than that of the IDE WE-IDE CE mode.

Then, the CA measurement was performed with the IDE strip. To mimic SMBG-type measurements, which measure the small amount of reduced mediator produced by the enzyme reaction in the large amount of oxidized mediator, the total concentrations of ferrocyanide and ferricyanide were kept constant (100 mM), and the concentration of ferrocyanide was changed from 0 to 10 mM.

**Figure 3:**
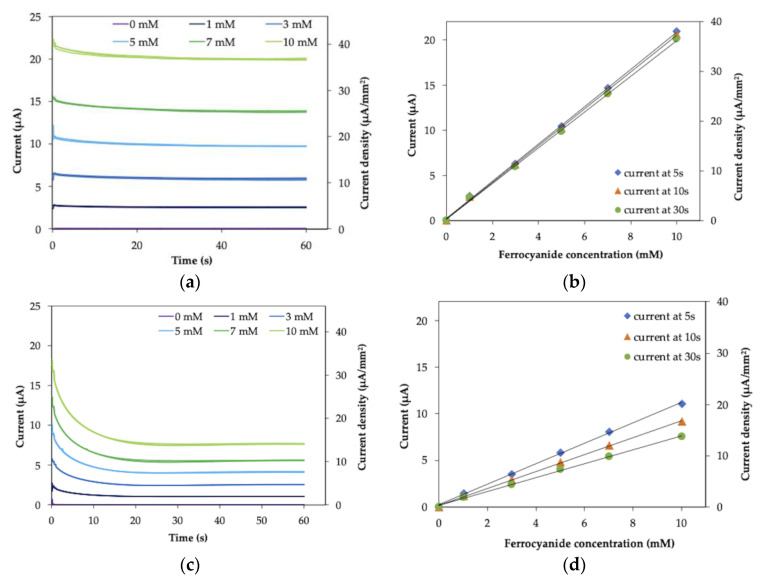
Response curves: (**a**) IDE WE-IDE CE mode; (**c**) IDE WE-plate CE mode, and the correlations between current and ferrocyanide concentration: (**b**) IDE WE-IDE CE mode; (**d**) IDE WE-plate CE mode, of chronoamperometry (CA) measurement of various concentrations of ferrocyanide mixed in ferricyanide with a total concentration of 100 mM. A potential of +0.4 V vs. Ag/AgCl was applied to observe the oxidation current of ferrocyanide. (**b**,**d**) are the plots of the resulting currents obtained at 5, 10 or 30 s after the potential application against the ferrocyanide concentration. Each measurement of ferrocyanide concentration was carried out in triplicate (*n* = 3).

The response curve and the correlations between current and ferrocyanide concentration measured in IDE WE-IDE CE mode are shown in Figure 3a,b. The response current reached a steady state immediately after the potential was applied and was dependent on the ferrocyanide concentration (Figure 3a). The ferrocyanide concentration-dependent current (Figure 3b) showed good linear correlation, and, remarkably, the slope of the calibration curve was independent of the time (5, 10 and 30 s) after application of the oxidation potential.

Figure 3c,d shows the response curve and the correlations between current and ferrocyanide in IDE WE-plate CE mode. The current gradually decreased until it reached a plateau after the application of the potential (Figure 3c). The ferrocyanide concentration-dependent current showed good linear correlations (Figure 3d); however, the slope of each correlation was dependent on time since the application of the potential (5 s, 10 s and 30 s). Furthermore, the observed current values in IDE WE-plate CE mode were smaller than those in IDE WE-IDE CE mode.

The slopes, y-intercept and linear regression coefficients of the CA measurement of ferrocyanide mixed in ferricyanide with IDE (Figure 3b,d) are summarized in Table S1. Since the slopes of the calibration curve are independent of the sampling time with IDE WE-IDE CE mode, the relative standard deviation (RSD) value of the slopes is small (2.8%). In contrast to this, with IDE WE-IDE plate mode, the slopes clearly depend on the sampling time, therefore, the RSD value (20%) was large compared to IDE WE-IDE CE mode. Since the y-intercept values are almost the same and the RSD values of the y-intercept are the same between two electrode configuration modes, the background current is not affected by the electrode configuration mode, and only the sampling time dependence of the slope was different between two modes.

The obtained current values and RSD values for each ferrocyanide concentration and each sampling time are summarized in Table S2. The RSD values are in same range between IDE WE-IDE CE mode and IDE WE-plate CE mode and are less than 2% for all ferrocyanide concentration except for 0 mM. Regarding the 0 mM, the current values are too small to discuss the RSD values. This observation also suggested that the dispersion of the obtained current values is not affected by the electrode configuration mode.

These results indicated that the superior characteristics of IDEs were demonstrated in IDE WE-IDE CE mode. This was achieved when one microelectrode of the IDE pair was used as the WE and the other as the CE, where the WE and the CE are placed in close distance (30 µm) with each other; thus, a non-diffusion limited, steady-state current was observed with both CV and CA measurements. The potential of the CE versus the RE during the CA measurement was monitored (Figure S1). The results indicated that the potential of CE was sufficient to reduce ferricyanide. This was consistent with the fact that the oxidation reaction (consumption of reduced species) occurred at the WE and the corresponding reduction reaction (production of reduced species) occurred at the CE. Consequently, a concentration gradient was formed between the WE and the CE, and the reduced-form mediator produced at the CE diffused to the WE. Thus, diffusion from the CE was dominant in the supply of the reduced-form mediator to the WE compared with diffusion from the bulk. Consequently, the reduced-form mediator was continuously supplied to the WE at a steady rate from the CE and was not diffusion limited; therefore, an immediate, steady-state and high electrical current was observed, as we expected. On the other hand, when the distance between the WE and the CE was large (IDE WE-plate CE mode), the supply of the reduced-form mediator to the WE was independent of the formation of the reduced form at CE but dependent on diffusion from the bulk, and a diffusion-limited current was observed. The distance between the WE and the CE was important only in the IDE WE-IDE CE mode, with which a large steady-state current was obtained.

### 2.2. Measurement of Fructosyl Lysine and GA with an IDE Enzyme Sensor Strip

IDE enzyme sensor strips for GA measurement were then constructed using FAOx as the enzyme and the Ru complex as the electron mediator.

**Figure 4:**
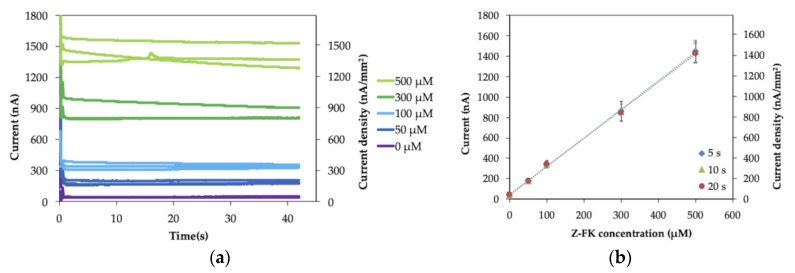
(**a**) Response curve and (**b**) calibration curve of Nα-Carbobenzyloxy-Nε-fructosyllysine (Z-FK) measurement with the IDE enzyme sensor strip in IDE WE*—*IDE CE mode (*n* = 3).

First, Nα-Carbobenzyloxy-Nε-fructosyllysine (Z-FK), the synthetic analog of ε-FK, was used as the substrate to evaluate the operational conditions of the IDE strip as the platform of a disposable GA sensor. The response curve and calibration curve of the Z-FK measurement are shown in Figure 4. The response curve (Figure 4a) indicates that the current immediately reached a plateau after the oxidation potential was applied, as was observed with the ferrocyanide/ferricyanide measurement (Figure 3a). The current values increased depending on the Z-FK concentration. Figure 4b shows the calibration curves, which are a correlation between the observed current and the Z-FK concentration, at 5, 10 or 30 s after the potential was applied. A good linear response was observed over the entire Z-FK concentration range (0*–*500 µM), with a linear regression coefficient of R^2^ = 0.999. The sensitivity and the slope of the calibration curve obtained at each period after potential application were identical and were not dependent on the sampling time. This was a significant difference and an advantage considering the reproducibility of the sensor signal compared with the enzyme sensor we previously reported using SPCE as the electrode [7], which showed dependency on the sampling time, and their slopes were different depending on the sampling time. The limit of detection (LOD), defined as the Z-FK concentration corresponding to the mean background current +3 standard deviations, was 1.2 µM. The LOD with the IDE was approximately 33 times lower than that with the SPCE, which showed an LOD of 40 µM Z-FK. The achieved sensitivity, 2.8 nA µM^−1^, was improved by 5.7 times using an IDE as the electrode compared with our previous achievement, which used an SPCE, with a sensitivity of 0.49 nA µM^−1^. The GA level is expressed as the proportion of the GA concentration to the total albumin concentration (%). The standard level of GA is from 11 to 16% according to the Japan Diabetes Society. At the current clinical site, the GA level is used as the glycemic control marker for the assessment of treatment effectiveness, but not for the diagnosis of diabetes. Therefore, there is no prescribed cut-off value to diagnose diabetes. The tentative target level for the treatment of diabetes patients is suggested as GA level < 20% by the Japanese Society for Dialysis Therapy. Considering that the average albumin concentration in the serum is 5 g/dL and the molecular weight of human serum albumin is 66.5 kDa, the calculated ε-FK concentrations are 83–120 µM for 11–16% GA and 150 µM for 20% GA. Therefore, the LOD and sensitivity are sufficient to detect and distinguish GA concentrations between healthy individuals and patients with diabetes.

The slopes, y-intercept and linear regression coefficients for Z-FK measurement (Figure 4b) are summarized in Table S3. Since the slope of the calibration curve is independent of sampling time, the RSD value of the slope (2.1%) was small, the same as for the ferrocyanide measurement (Table S1).

The obtained current values and RSD values for each Z-FK concentration and each sampling time are summarized in Table S4. Comparing with ferrocyanide measurement (Table S2), the RSD values are larger and dispersed for the Z-FK measurement with IDE enzyme sensor strip (0.8–12%). This large RSD values might be due to the lot-to-lot variations of enzyme sensor strip and the measurement process. To prepare the enzyme sensor strip, we put the mixture containing enzyme and mediator on the IDE, dried them, and then applied the spacer and cover to make the capillary on the electrode. It is possible that there are lot-to-lot variations of the prepared sensor strip due to inconsistency of performance. In addition, in the measurement process, after injecting the sample solution in the capillary on the sensor strip, the time and process are required to dissolve the dried enzyme and mediator on the enzyme sensor strips with the sample solution. This dissolving process might affect the homogeneity of the sequential reaction of the substrate oxidation with the enzyme and reduction of the mediator and might lead to the dispersion of the sensor signal. These points might be the reason why the RSD values of the Z-FK measurement with enzyme sensor strips are larger compared with the ferrocyanide solution measurement with bare IDE (Table S2). However, in the practical application, the signals with good reproducibility are expected with manufactured enzyme sensor strips.

**Figure 5:**
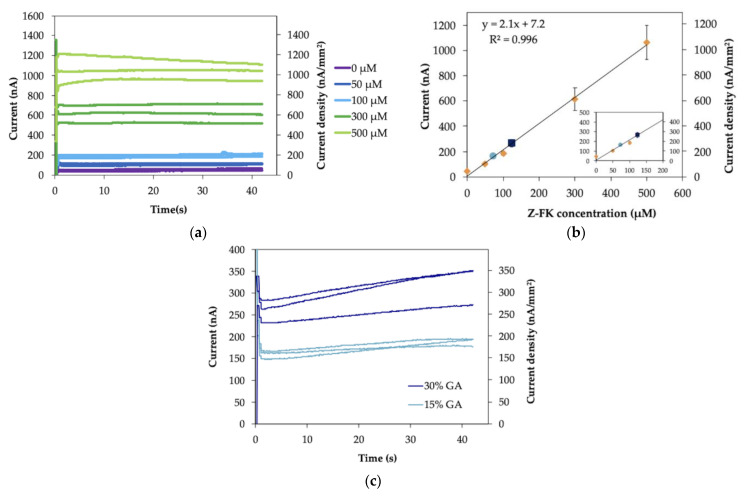
(**a**) Response curve and (**b**) calibration curve of measurement of various concentrations of Z-FK contained in protease-digested nonglycated albumin sample with the IDE enzyme sensor strip in IDE WE-IDE CE mode (*n* = 3). (**c**) Response curve of protease-digested glycated albumin (GA) sample measurement with the IDE enzyme sensor strip in IDE WE-IDE CE mode (*n* = 3). The current values obtained from protease-digested GA samples were plotted on the calibration curve (**b**) as circles (15% GA) and squares (30% GA).

Then, protease-digested GA samples were measured with an IDE enzyme sensor strip. To obtain a calibration curve of the protease-digested GA sample, several concentrations of Z-FK were spiked into the protease-digested nonglycated albumin sample, and their response was analyzed. The response curve is shown in Figure 5a. The calibration curve of the current at 5 s after application of the potential versus Z-FK concentration is plotted in Figure 5b. The linear range was 0*–*500 µM Z-FK with a linear regression coefficient of R^2^ = 0.996, and the LOD was 25 µM in the presence of protease digestion materials. The presence of several molecules derived from protease-digested materials, such as amino acids or peptides derived from albumin, may alter pH, ionic strength, and/or viscosity of sample solution, and affect the sensitivity and LOD of the sensor. The presence of these derivatives might have a negative impact on the enzyme activity and/or electrochemical reaction by affecting the diffusion of the mediator. Consequently, the sensitivity and LOD of the sensor might be changed in the presence of protease-digested non-glycated albumin. In addition, the target detection range of ε-FK concentration, which is calculated from physiological level of GA, is around the hundreds µM level. Considering this target range of GA measurement, the sensor will still be sensitive enough to cover the physiological range of the ε-FK concentration, even in the protease-digested sample, where the LOD increases from 1.2 µM to 25 µM. Therefore, we concluded that the sensor will be used in the protease-digested sample; even the LOD is drastically increased in the presence of derivatives of protease digestion. In this study, two different GA samples were used, 15 and 30%. The GA level is the indicator of glycemic control and at the current clinical site, the GA value is used for the assessment of treatment effectiveness, but not for the diagnosis of diabetes. Therefore, there is no prescribed cut-off value to diagnose diabetes. According to the Japan Diabetes Society, the GA level of a healthy subject is 11*–*16%. The Japanese Society for Dialysis Therapy suggested that the therapeutic target GA level of diabetic patients should be <20%. This indicated that when diabetic patient under the treatment shows a GA value higher than 20%, such as 30%, a revision of treatment will be required. Therefore, the samples with GA values of 15 or 30% represent the one for the healthy subject or the one for the diabetic patient whose glycemic level was not adequately controlled, respectively. Figure 5c shows the sensor responses toward two concentrations (15 and 30%) of protease-digested GA samples. An immediately plateauing current was obtained, and the current value was dependent on the GA level. The RSD values of obtained currents for 15 and 30% GA samples were 6 and 10%, respectively. These values were in the same range with those for the Z-FK measurement (Table S4). Therefore, the reproducibility of the protease-digested GA sample measurement might also be affected by lot-to-lot variations of sensor strip preparation and the dissolving process of enzyme and mediator dried on the sensor strip by sample solution in the measurement process. The ε-FK concentrations of two protease-digested GA samples were determined to be 79 ± 4 µM and 136 ± 17 µM for 15 and 30% GA, respectively, based on the calibration curve obtained with Z-FK in the presence of protease-digested albumin (Figure 5b). The difference between the current values obtained with 15% GA and 30% GA was 104 nA, which was more than three-fold greater than the results obtained in the enzyme sensor with an SPCE (34 nA). These results indicated that a high-resolution measurement was successfully achieved for GA measurement when an IDE was employed as the electrode in the IDE WE-IDE CE mode.

Several studies about the electrochemical biosensor for GA measurement have been reported using enzymes, antibody, aptamer or peptide as the biorecognition molecules and based on amperometry, electrochemiluminescence (ECL), impedance, voltammetry or field effect transistor (FET) as sensing principles [7,16,17,18,19,20,21,22,23]. These studied are summarized in Table 1. Among these studies, this study showed the smallest sample volume and shortest waiting time, which is the period before starting electrochemical measurement after the sample solution was added on the electrode. Considering the time taken to perform the electrochemical measurement among these studies, ECL, square wave voltammetry (SWV), impedance and FET required several minutes to obtain the resulting signal as these need to sweep the apply potential or change the potential with various frequencies. On the other hand, with the amperometry method, especially in this study, the resulting signal was obtained within 5 sec after starting the electrochemical measurement by potential application. Therefore, the developed enzyme sensor using IDE in this study showed superior characteristic compared with other reported sensing systems.

Not only dual potentiometry-based amperometric sensors have been reported using IDEs but also various types of electrochemical biosensors. Regarding chronoamperometric biosensors, antibodies [8,9,10], aptamers [11], peptides [12] and enzymes [13] have been used as recognition molecules, and they are based on dual potentiometry. Regarding impedimetric biosensors using IDE, antibodies [24,25,26,27,28] and aptamers [29] have been used as the primary molecular recognition elements, as affinity-based detection requires strong binding and selective biomolecules for impedance measurement. With impedimetric biosensors, measurement is based on the access of a redox probe to the electrode surface, and changes occur when an insulating layer is produced with the recognition element/antigen complex, which reduces the transfer of electrons. As a result, an increase in charge transfer resistance (Rct) occurs. Regarding capacitive biosensors, antibodies [30,31,32,33], aptamers [34,35], and affimers [36] have been used as recognition elements. Capacitive biosensors rely on changes occurring within the electrical double layer (EDL), where the thickness is changed according to binding of a target to a recognition element on the electrode. Changes in the target concentration correspond to changes occurring at the EDL. Nondual potentiometry-based amperometric sensors using enzymes have also been reported [37,38]. Sharma et al. immobilized an enzyme–glucose oxidase [37] or cholesterol oxidase [38] on one microelectrode of an IDE pair and measured the substrate concentration in the presence of ferricyanide as the electron mediator by applying the oxidation potential to both the WE1 and WE2. In this measurement, the reduced-form mediator produced by the enzyme reaction (oxidation of the substrate) was oxidized at both WE1 and WE2. The authors applied only the oxidation potential to the WEs; however, since the distance between the WEs and CE was large, the current response time was slightly slow. On the other hand, in this study, by focusing on the distance between the WE and the CE, and by using one IDE of a pair as the WE and the other IDE as the CE, a highly sensitive endpoint assay-type disposable enzyme sensor for GA was developed. Furthermore, the application of the IDE in the IDE WE-IDE CE mode should not be limited to GA measurement. By using other enzymes for other target molecules, highly sensitive and reproducible disposable enzyme sensors are expected based on an IDE as the platform electrode.

## 3. Materials and Methods

### 3.1. Materials and Apparatus

Disposable 2-electrode and 4-electrode interdigitated electrode (IDE) strips were kindly supplied by Tanaka Kikinzoku Kogyo K.K. (Tokyo, Japan). The 2-electrode strips consisted of two interdigitated Au electrodes with a band gap and width of 30 µm each, while the 4-electrode strips additionally had two macroplate electrodes (0.30 mm^2^ and 0.43 mm^2^ each) (Figure 1a). Fructosyl amino acid oxidase (FAOx) was prepared according to Kouzuma et al. [6]. Nα-Carbobenzyloxy-Nε-fructosyllysine (Z-FK) was prepared according to Hashiba [39]. Protease-digested albumin and protease-digested glycated albumin were prepared as previously reported [7]. Potassium hexacyanoferrate (III) (K_3_[Fe(CN)_6_], ferricyanide) and potassium hexacyanoferrate (II) (K_4_[Fe(CN)_6_], ferrocyanide) were purchased from Kanto Chemical Co., Inc. (Tokyo, Japan), and hexaammineruthenium (III) chloride (Ru(NH_3_)_6_Cl_3_, Ru-complex) was purchased from Sigma-Aldrich (St. Louis, MO, USA). All other chemicals were of analytical grade. Electrochemical experiments were performed using an HSV-100 potentiostat (Hokuto Denko Co., Tokyo, Japan).

### 3.2. Electrode Characterization by Ferrocyanide/Ferricyanide Redox Couple Measurement

Ag/AgCl paste was dried onto an Au plate at the top of a 4-electrode-IDE strip to be used as a reference electrode. First, cyclic voltammetry (CV) measurements were performed. A drop (5 µL) of a mixture of 1 mM ferrocyanide and 9 mM ferricyanide (the total concentration of ferrocyanide and ferricyanide was 10 mM) in 100 mM KCl was deposited on the electrode area. CV measurements were performed with the potential range from +0.19 to +0.6 V vs. Ag/AgCl at 10 mV/sec. Then, chronoamperometry (CA) measurements were also performed. A drop (5 µL) of a mixture of various concentrations of ferrocyanide (0*–*10 mM) along with ferricyanide, with the total concentration of ferrocyanide and ferricyanide being 100 mM, in 100 mM KCl was deposited on the electrode strip. A potential of +0.4 V vs. Ag/AgCl was applied, and the current was recorded over 60 s. Both CV and CA measurements were performed either in IDE WE-IDE CE mode, where one microelectrode of the IDE pair was used as the WE and the other as the CE (Figure 1b), or IDE WE-plate CE mode, where one microelectrode of the IDE pair was used as the WE and the external plate electrode on the IDE strip was used as the CE (Figure 1c).

### 3.3. Preparation and Characterization of IDE Enzyme Sensor Strip for GA Measurement

A volume of 0.8 µL of a solution of 60 U/mL FAOx (optimized concentration, see Figures S3 and S4), 300 mM Ru-complex (optimized concentration, see Figures S5 and S6), and 0.25% sucrose in 100 mM PPB, pH 8.0, was dropped onto 2-electrode-IDE strips and dried at 25 °C. Then, a spacer and a cover were attached to the electrodes. All sensors were used immediately in this study.

For the measurement, 0.8 µL of samples of different concentrations of Z-FK was injected into the spacer layer of the enzyme sensor strips. The potential of +0.1 V vs. Au was applied 60 s after sample injection, and the current was observed. Additionally, various concentrations of Z-FK contained in protease-digested, nonglycated albumin and protease-digested GA samples were measured with the same method.

## 4. Conclusions

In this study, we focused on the superior characteristics of IDEs when they are used as an alternative platform technology for disposable enzyme sensor strips for GA measurement, a glycemic control marker for diabetes. We demonstrated that by using a pair of IDEs as the WE and the CE, the distance between the WE and CE was relatively small, and a time-independent, steady-state and large current was achieved. Furthermore, the obtained current was dependent on the concentration of only the reduced-form mediator in the presence of the oxidized-form mediator when the oxidation potential was applied to the WE. The prepared IDE enzyme sensor strip for GA measurement showed a large, steady current, which led to higher sensitivity than that of the SPCE in our previous study. The measurements of protease-digested GA samples were also demonstrated to have high sensitivity with the IDE. The novel application of IDE for the development of highly sensitive and reproducible endpoint assay-type enzyme sensors has been demonstrated, and further application of IDE as a platform for various enzyme-based sensors is expected.

## Figures and Tables

**Table 1:** Comparison of reported electrochemical biosensors for GA measurement.

Biorecognition Molecule		Target Molecule	Electrochemical Principle	Electrode	Sample Volume	Waiting Time ^1^	Measurement Range	Ref.
Enzyme	FAOx	ε-FK	Amperometry	Disposable IDE	0.8 µL	1 min	50–500 µM (LOD: 1.2 µM)	This study
	FN6K	GA	Amperometry	Disposable SPCE	8 µL	10 min	20–100 µM	[16]
	FAOx	ε-FK	Amperometry	Disposable SPCE	1.3 µL	1 min	50–500 µM (LOD: 40 µM)	[7]
	FAOx	ε-FK	ECL	Disposable SPCE	100 µL	1 min	0.1–2 µM (LOD: 0.1 µM)	[17]
Anti-HSA antibody		GA	Impedance	IDE	No information	15 min	1–400 ng mL^−1^	[18]
				Au electrode	No information	15 min	0–15.7 %	[19]
Aptamer		GA	SWV	SPCE	No information	40 min	2 × 10^−6^–16 mg mL^−1^ (LOD: 2 × 10^−6^ mg mL^−1^)	[20]
Aptamer		GA	SWV	rGO/Au NP	40 µL	30 min	2–10 µg mL^−1^ (LOD: 0.07 µg mL^−1^)	[21]
Aptamer		GA	FET	Au coated ZnO	>60 µL	Few minutes	77–343 µg mL^−1^	[22]
HSA-specific peptide, Boronic acid-modified DHFR		GA	SWV	PEDOT electrode	100 µL	15 min	1–1000 nM (LOD: 1 nM)	[23]

^1^ The period before starting electrochemical measurement after the sample solution was added on the electrode. Abbreviations; FAOx: fructosyl amino acid oxidase; FN6K: Fructosamine 6-kinase; HSA: human serum albumin; DHFR: dihydrofolate reductase; ε-FK: ε-fructosyl lysine; GA: Glycated albumin; ECL: electrochemiluminescence; SWV: square wave voltammetry; FET: field effect transistor; IDE: interdigitated electrode; SPCE: screen-printed carbon electrode; rGO: reduced graphene oxide; Au NP: gold nano particle; PEDOT: poly(3,4-ethylenedioxythiophene); LOD: limit of detection.

## Data Availability

Not applicable.

## Supplementary Materials

The following are available online, S1: Characteristic parameters for CA measurements of ferrocyanide with IDE (Table S1, S2), S2: Measurement of the potential of the counter electrode during the chronoamperometry (CA) measurement using the interdigitated array electrode (IDE) in IDE WE-IDE CE mode (Figure S1, S2), S3; Parameters of CA measurements of Z-FK with IDE enzyme sensor strip (Table S3, S4), S4; Optimization of the FAOx concentration for the enzyme sensor for the glycated albumin (GA) measurement (Figure S3, S4, Table S5), S5; Optimization of the mediator concentration for the enzyme sensor for the glycated albumin (GA) measurement (Figure S5, S6, Table S6).
