# Supplementary material for: Development of an Interdigitated Electrode-Based Disposable Enzyme Sensor Strip for Glycated Albumin Measurement
Source: Molecules. 2021 Jan 31;26(3):734. doi: 10.3390/molecules26030734 (PMC7866809; doi:10.3390/molecules26030734)
Supplement: Supplementary file 1 [file molecules-26-00734-s001.pdf]

# Supplementary information

# Development of an Interdigitated Electrode-Based Disposable Enzyme Sensor Strip for Glycated Albumin Measurement

Mika Hatada[1]†, Noya Loew[1]†, Junko Okuda-Shimazaki[1], Mukund Khanwalker[1], Wakako Tsugawa[2], Ashok Mulchandani[3], Koji Sode[1,*]

[1] Joint Department of Biomedical Engineering, The University of North Carolina at Chapel Hill and North Carolina State University, Chapel Hill, NC 27599, USA; mikah@email.unc.edu (M.H.); noya-loew@rs.tus.ac.jp (N.L.); jokudas@email.unc.edu (J.O-S.); mukund@live.unc.edu (M.K.); ksode@email.unc.edu (K.S.)

[2] Department of Biotechnology and Life Science, Graduate School of Engineering, Tokyo University of Agriculture and Technology, 2-24-16 Naka-cho, Koganei, Tokyo, 184-8588, Japan; tsugawa@cc.tuat.ac.jp (W.T.)

[3] Department of Chemical and Environmental Engineering, University of California, Riverside, CA 92521, USA; adani@engr.ucr.edu (A.M.)

* Correspondence: ksode@email.unc.edu; Tel.: +1-919-966-3550

†These authors contributed equally

## S1. Characteristic parameters for CA measurements of ferrocyanide with IDE

The slopes, y-intercept and linear regression coefficients of chronoamperometry (CA) measurement of ferrocyanide mixed in ferricyanide using IDE (corresponding to Figure 3b and 3d in main manuscript) are summarized in Table S1. Also, the obtained current values and relative standard deviation (RSD) values for each ferrocyanide concentration and each sampling time are summarized in Table S2.

**Table S1**. Characteristic parameters of CA measurements of ferrocyanide with IDE

| Configuration Mode | Sampling time [s] | Slope (sensitivity) [μA mM$^{-1}$] | RSD of slope [%] | y-intercept [μA] | RSD of y-intercept [%] | $R^2$ |
|---|---|---|---|---|---|---|
| IDE WE – IDE CE mode | 5 | 2.1 | 2.8 | 0.20 | 14 | 0.999 |
| | 10 | 2.1 | | 0.18 | | 0.999 |
| | 30 | 2.0 | | 0.15 | | 0.999 |
| IDE WE – plate CE mode | 5 | 1.1 | 20 | 0.22 | 14 | 0.999 |
| | 10 | 0.9 | | 0.17 | | 0.999 |
| | 30 | 0.74 | | 0.18 | | 0.998 |

**Table S2.** Current values and RSD values for each sampling time and each ferrocyanide concentration (a: IDE WE-IDE CE mode, b: IDE WE-plate CE mode)

**Table S2a**

| | 5 s | | 10 s | | 30 s | |
|---|---|---|---|---|---|---|
| Ferrocyanide conc. [mM] | Current [μA] | RSD [%] | Current [μA] | RSD [%] | Current [μA] | RSD [%] |
| 0 | $8.3x10^{-3}±3.7x10^{-3}$ | 45 | $7.9x10^{-3}±4.0x10^{-3}$ | 51 | $5.7x10^{-3}±2.8x10^{-3}$ | 48 |
| 1 | $2.7±3.4$ $x10^{-2}$ | 1.3 | $2.6±3.5$ $x10^{-2}$ | 1.3 | $2.5±4.2$ $x10^{-2}$ | 1.7 |
| 3 | $6.2±7.4$ $x10^{-2}$ | 1.2 | $6.1±8.0$ $x10^{-2}$ | 1.3 | $5.9±8.6$ $x10^{-2}$ | 1.5 |
| 5 | $1.0x10±9.2$ $x10^{-2}$ | 0.88 | $1.0x10±7.0$ $x10^{-2}$ | 0.69 | $9.8±5.1$ $x10^{-2}$ | 0.52 |
| 7 | $1.5x10±4.5$ $x10^{-2}$ | 0.31 | $1.4x10±3.6$ $x10^{-2}$ | 0.25 | $1.4x10±7.5$ $x10^{-2}$ | 0.54 |
| 10 | $2.1x10±1.7$ $x10^{-1}$ | 0.80 | $2.1x10±1.3$ $x10^{-1}$ | 0.62 | $2.0x10±6.2$ $x10^{-2}$ | 0.31 |

**Table S2b**

| | 5 s | | 10 s | | 30 s | |
|---|---|---|---|---|---|---|
| Ferrocyanide conc. [mM] | Current [μA] | RSD [%] | Current [μA] | RSD [%] | Current [μA] | RSD [%] |
| 0 | $8.4x10^{-3}±2.3x10^{-3}$ | 28 | $6.2x10^{-3}±1.9x10^{-3}$ | 30 | $4.4x10^{-3}±1.2x10^{-3}$ | 28 |
| 1 | $1.5±1.9$ $x10^{-2}$ | 1.3 | $1.2±1.5$ $x10^{-2}$ | 1.2 | $1.0±6.8$ $x10^{-3}$ | 0.67 |
| 3 | $3.5±1.6$ $x10^{-2}$ | 0.46 | $2.9±1.8$ $x10^{-2}$ | 0.64 | $2.4±5.7$ $x10^{-3}$ | 0.23 |
| 5 | $5.8±4.3$ $x10^{-2}$ | 0.75 | $4.7±2.6$ $x10^{-2}$ | 0.55 | $4.0±3.7$ $x10^{-2}$ | 0.92 |
| 7 | $8.0±2.1$ $x10^{-2}$ | 0.26 | $6.6±1.3$ $x10^{-2}$ | 0.19 | $5.4±1.0$ $x10^{-1}$ | 1.9 |
| 10 | $1.1x10±5.3$ $x10^{-2}$ | 0.48 | $9.1±1.6$ $x10^{-2}$ | 0.18 | $7.5±1.0$ $x10^{-1}$ | 1.4 |

## S2. Measurement of the potential of the counter electrode during the chronoamperometry (CA) measurement using the interdigitated array electrode (IDE) in IDE WE-IDE CE mode

The potential of the counter electrode (CE) against the reference electrode (RE) during the chronoamperometry measurement of ferrocyanide/ferricyanide in the IDE WE-IDE CE mode was measured.

A drop (5 μL) of a mixture of various concentrations of ferrocyanide (0 - 10 mM) along with ferricyanide, with the total concentration of ferrocyanide and ferricyanide being 100 mM in 100 mM KCl was deposited on the electrode area. A potential of +0.4 V vs Ag/AgCl was applied, and the current was recorded over 60 s. At the same time, the potential of the CE against the RE was monitored. Cyclic voltammetry (CV) measurements of 5 mM ferricyanide in 100 mM KCl were also performed to confirm the redox potential of the ferrocyanide/ferrocyanide couple.

As a result of the CA measurement, a steady-state current was observed (Figure S1a), and the current value increased linearly depending on the ferrocyanide concentration (Figure S1b), corresponding to Figure 3a and 3b in the main text. Regarding the potential of the CE, the potentials against the RE were stable during the CA measurement (Figure S1c) and were approximately -20 to -40 mV. Since the redox potential of ferricyanide/ferrocyanide is approximately +100 mV vs Ag/AgCl according to the CV measurement (Figure S2), it was suggested that the potential of CE vs RE was kept at a sufficient level to reduce ferricyanide to ferrocyanide. Therefore, the reduction reaction of ferricyanide might occur at the CE during the CA measurement, where the oxidation reaction of ferrocyanide occurred at the WE by the application of +400 mV vs Ag/AgCl against the WE.

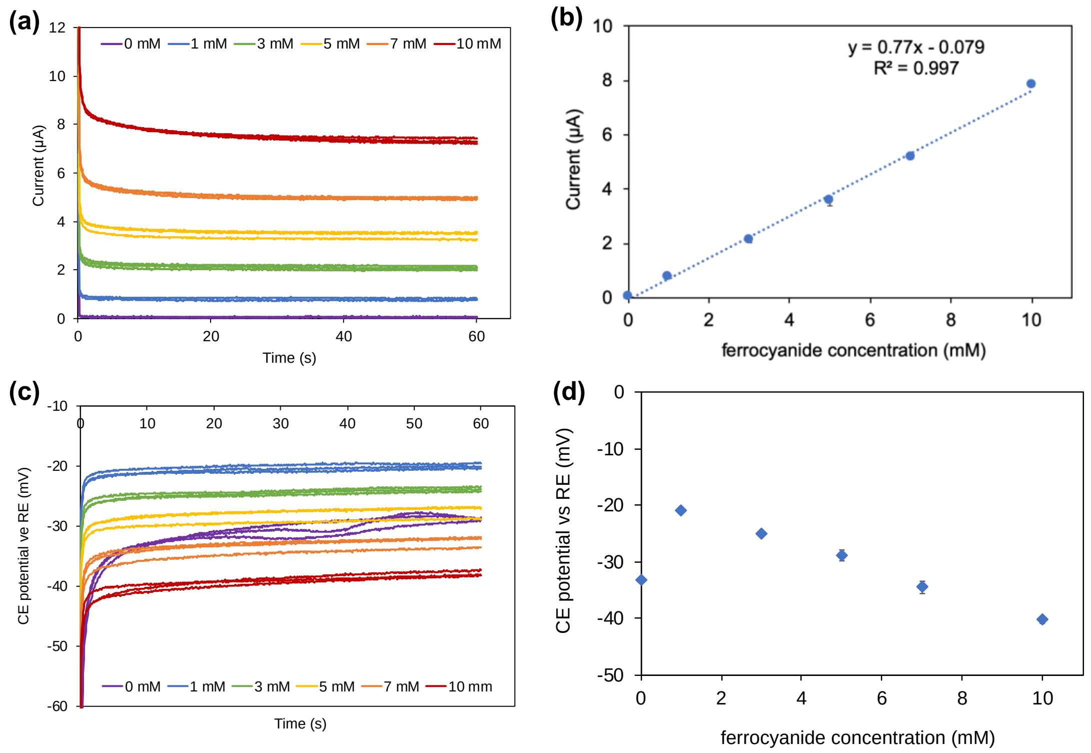


**Figure S1.** The response curve (a) and the calibration curve (b) of CA measurement of various concentrations of ferrocyanide mixed in ferricyanide with a total concentration of 100 mM (n=3). The potential of +0.4 V vs. Ag/AgCl was applied to observe the oxidation current of ferrocyanide. At the same time, the potential of the CE against the RE was monitored, and the time course and the calibration curve are shown as (c) and (d), respectively.

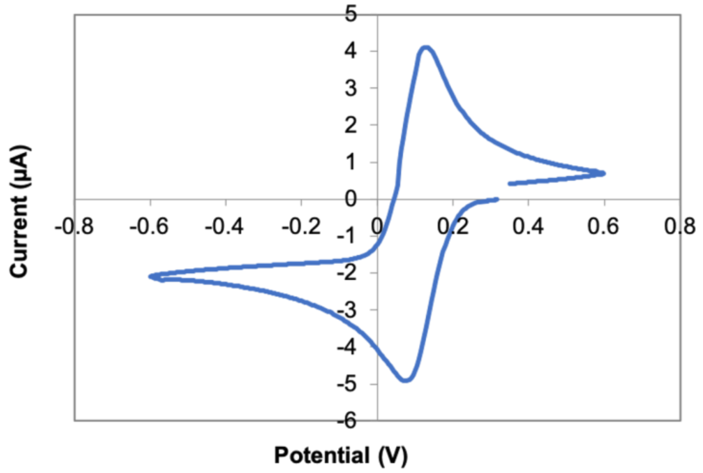


**Figure S2.** The cyclic voltammogram of ferricyanide.

## S3. Parameters of CA measurements of Z-FK with IDE enzyme sensor strip

The slopes, y-intercept and linear regression coefficients for Z-FK measurement with IDE enzyme sensor strip (corresponding to Figure 4b in main text) are summarized in Table S3. Also, the obtained current values and RSD values for each Z-FK concentration and each sampling time are summarized in Table S4.

**Table S3.** Characteristic parameters of CA measurements of Z-FK with IDE enzyme sensor strip

| **Configuration Mode** | **Sampling time [s]** | **Slope (sensitivity) [μA $mM^{-1}$]** | **RSD of slope [%]** | **y-intercept [μA]** | **RSD of y-intercept [%]** | **$R^2$** |
|---|---|---|---|---|---|---|
| IDE WE – IDE CE mode | 5 | 2.8 | 2.1 | 44 | 4.6 | 0.999 |
| | 10 | 2.8 | | 45 | | 0.999 |
| | 20 | 2.7 | | 48 | | 0.999 |

**Table S4.** Current values and RSD values for each sampling time and each Z-FK concentration

| | **5 s** | | **10 s** | | **20 s** | |
|---|---|---|---|---|---|---|
| Z-FK conc. [μM] | Current [nA] | RSD [%] | Current [nA] | RSD [%] | Current [nA] | RSD [%] |
| 0 | $4.2x10±1.8$ | 4.3 | $4.2x10±3.4x10^{-1}$ | 0.8 | $4.4x10±1.0$ | 2.5 |
| 50 | $1.8x10^2±2.2x10$ | 12 | $1.8x10^2±2.1x10$ | 12 | $1.8x10^2±1.9x10$ | 11 |
| 100 | $3.4x10^2±3.7x10$ | 11 | $3.4x10^2±3.3x10$ | 9.6 | $3.4x10^2±2.8x10$ | 8.1 |
| 300 | $8.6x10^2±1.0x10^2$ | 12 | $8.6x10^2±9.4x10^1$ | 11 | $8.5x10^2±8.1x10^1$ | 9.5 |
| 500 | $1.5x10^3±1.0x10^2$ | 7.4 | $1.4x10^3±1.0x10^2$ | 7.1 | $1.4x10^3±1.0x10^2$ | 7.0 |

## S4. Optimization of the FAOx concentration for the enzyme sensor for the glycated albumin (GA) measurement

The concentration of enzyme, FAOx for the electrochemical GA measurement was optimized by the Z-FK measurement. A volume of 0.8 μL of a solution containing various concentrations of Z-FK (0, 50, 100, 300, and 500 μM), 100 mM Ru complex and various concentration of FAOx (1.2, 2.4, 6, 9, 12, 24, 48, 60, 120, 160, 240 U/mL) in 100 mM potassium phosphate buffer (pH 8.0) was injected into the spacer layer of the interdigitated array electrode (IDE) strip. The potential of +0.1 V vs. Au was applied 60 s after the sample injection, and the current was observed.

The time courses of Z-FK measurement with the FAOx concentrations are shown in Figure S3. Additionally, the calibration curves are shown in Figure S4a. The slopes, y-intercepts and linear regression coefficients of the calibration curves are summarized in TableS5. Figure S4b is the plot of the sensitivity (slope) and the y-intercept (background) of the calibration curves of the Z-FK measurement against the FAOx concentration. Both the sensitivity and the background current increased depending on the FAOx concentration with the 12 U/mL FAOx concentration. They were almost the same value when using a FAOx concentration over 12 U/mL. (Figure S4b). Therefore, it was suggested that over 12 U/mL FAOx is required to oxidize all the substrate within the 60 sec incubation time, and analytical performance was improved by using over 12 U/mL of enzyme by increasing the sensitivity. Considering that during the preparation process of enzyme sensor strip, the inactivation of FAOx may occur, the excess amount of FAOx, 60 U/mL was used as the optimized concentration for FAOx.

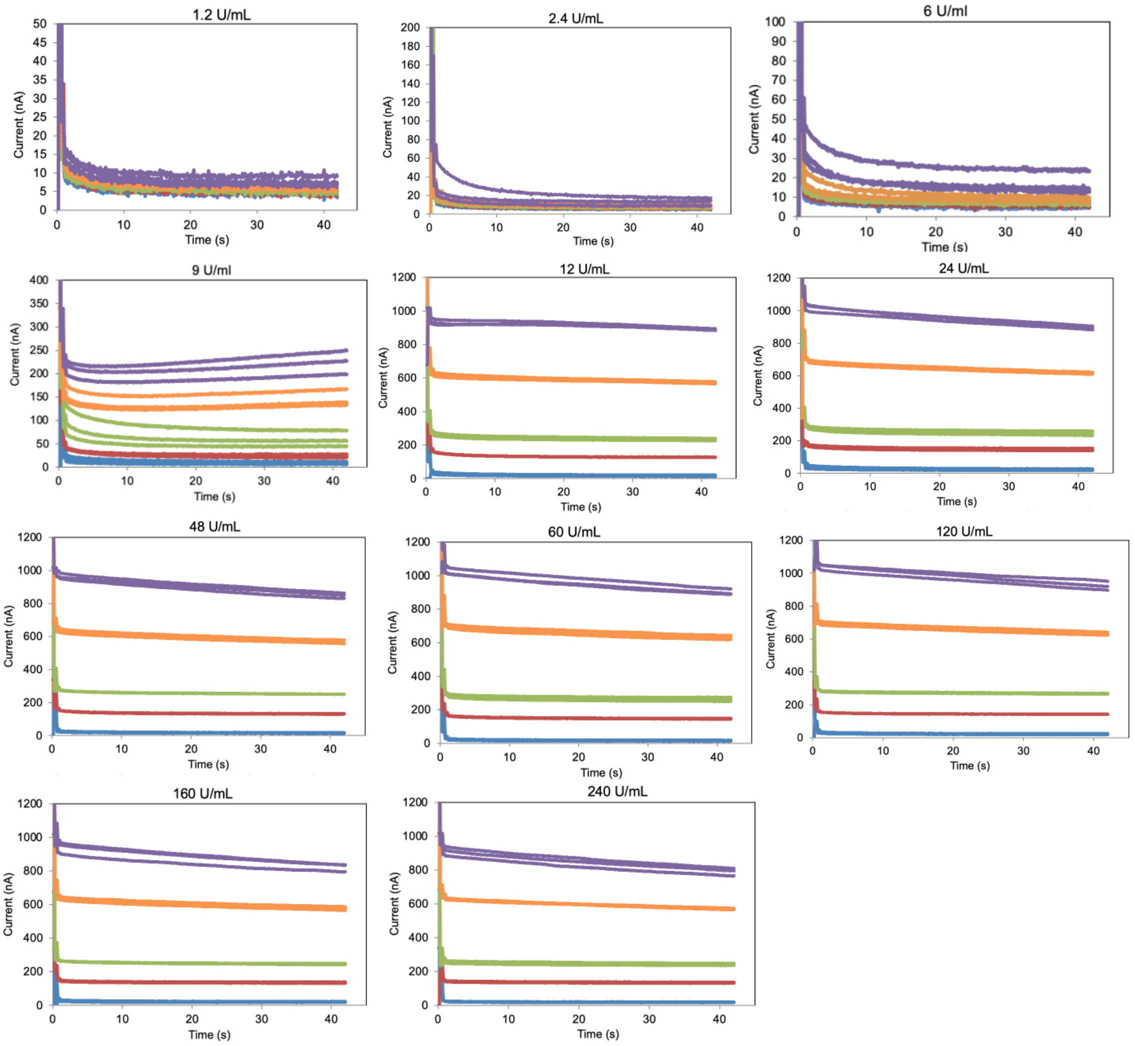


**Figure S3.** The response curves of Z-FK measurements with various concentrations of FAOx.

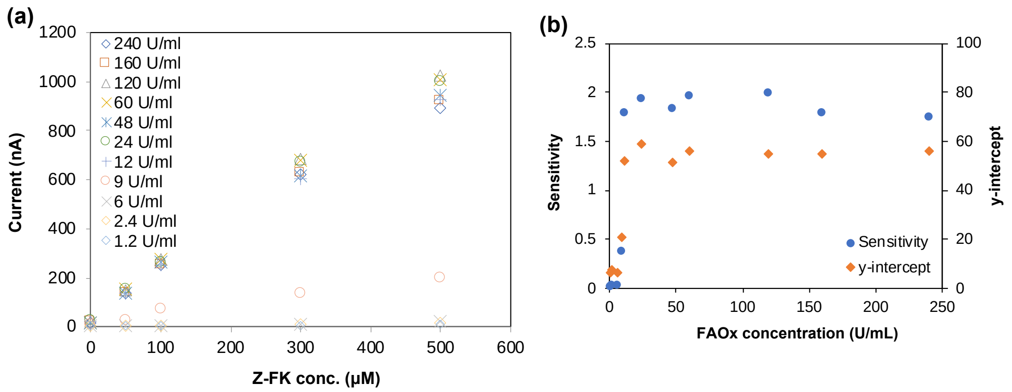


**Figure S4.** (a) The calibration curves of Z-FK measurements with various concentrations of FAOx. (b) Plot of the sensitivity (slope) and the y-intercept of the calibration curve of the Z-FK measurement against the FAOx concentration.

**Table S5**. Characteristic parameters of CA measurements of Z-FK with various concentration of FAOx

| Configuration Mode | FAOx conc. [U mL$^{-1}$] | Slope (sensitivity) [μA mM$^{-1}$] | RSD of slope [%] | y-intercept [μA] | RSD of y-intercept [%] | $R^2$ |
|---|---|---|---|---|---|---|
| | 1.2 | 0.0073 | | 6.3 | | 0.971 |
| | 2.4 | 0.026 | | 7.5 | | 0.974 |
| | 6 | 0.036 | | 6.1 | | 0.919 |
| | 9 | 0.37 | | 21 | | 0.979 |
| | 12 | 1.8 | | 52 | | 0.997 |
| IDE WE – IDE CE mode | 24 | 1.9 | 73 | 59 | 59 | 0.996 |
| | 48 | 1.8 | | 51 | | 0.996 |
| | 60 | 2.0 | | 56 | | 0.994 |
| | 120 | 2.0 | | 55 | | 0.995 |
| | 160 | 1.8 | | 55 | | 0.994 |
| | 240 | 1.7 | | 56 | | 0.991 |

## S5. Optimization of the mediator concentration for the enzyme sensor for the glycated albumin (GA) measurement

The concentration of hexaammineruthenium(III) chloride (the Ru complex) as the mediator for the electrochemical GA measurement was optimized by the Z-FK measurement. A volume of 0.8 μL of a solution containing various concentrations of Z-FK (0, 50, 100, 300, and 500 μM), 60 U/mL FAOx and various concentrations of Ru complex (1, 10, 50, 100, 150, 200, 300, and 400 mM) in 100 mM potassium phosphate buffer (pH 8.0) was injected into the spacer layer of the interdigitated array electrode (IDE) strip. The potential of +0.1 V vs. Au was applied 60 s after the sample injection, and the current was observed.

The time courses of Z-FK measurement with the various Ru complex concentrations are shown in Figure S5. Additionally, the calibration curves are shown in Figure S6a. The slopes, y-intercepts and linear regression coefficients of the calibration curves are summarized in TableS6. Figure S6b is the plot of the sensitivity (slope) and the y-intercept (background) of the calibration curves of the Z-FK measurement against the Ru complex concentration. Both the sensitivity and the background current increased depending on the Ru complex concentration with the 200 mM Ru complex concentration. They were almost the same value when using a Ru complex concentration over 200 mM. (Figure S6b). It was suggested that the concentration of the Ru complex required no limits to the sequential reaction of substrate oxidation with the enzyme, in addition, the reduction of the mediator was over 200 mM. Therefore, in this study, 300 mM Ru complex was used as the optimized mediator condition.

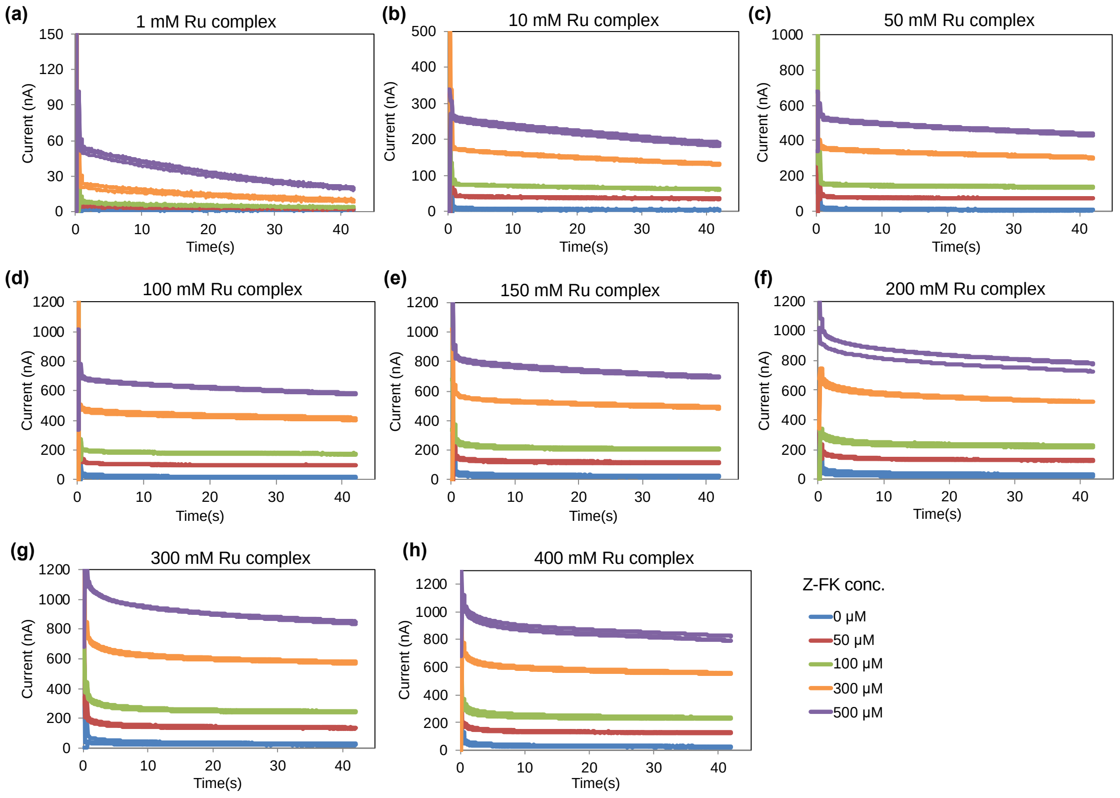


**Figure S5.** The response curves of Z-FK measurements with various concentrations of the Ru complex. (a) 1 mM, (b) 10 mM, (c) 50 mM, (d) 100 mM, (e) 150 mM, (f) 200 mM, (g) 300 mM and (h) 400 mM Ru complex.

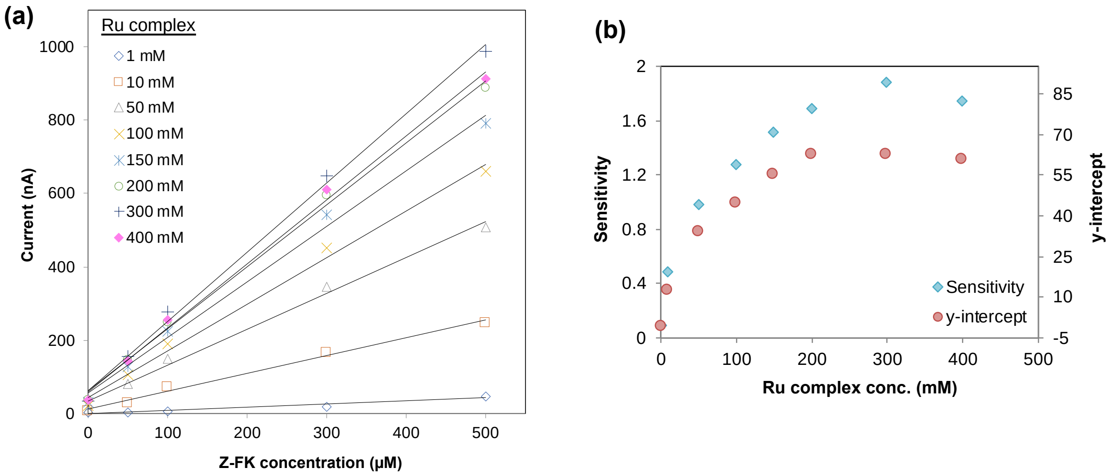


**Figure S6.** (a) The calibration curves of Z-FK measurements with various concentrations of the Ru complex. (b) Plot of the sensitivity (slope) and the y-intercept of the calibration curve of the Z-FK measurement against the Ru complex concentration.

**Table S6.** Characteristic parameters of CA measurements of Z-FK with various concentration of Ru complex

| Configuration Mode | Ru complex conc. [mM] | Slope (sensitivity) [μA mM$^{-1}$] | RSD of slope [%] | y-intercept [μA] | RSD of y-intercept [%] | $R^2$ |
|---|---|---|---|---|---|---|
| IDE WE – IDE CE mode | 1 | 0.088 | 53 | 0.84 | 58 | 0.958 |
| | 10 | 0.48 | | 12 | | 0.991 |
| | 50 | 0.98 | | 34 | | 0.992 |
| | 100 | 1.3 | | 44 | | 0.993 |
| | 150 | 1.5 | | 55 | | 0.993 |
| | 200 | 1.7 | | 62 | | 0.996 |
| | 300 | 1.9 | | 63 | | 0.996 |
| | 400 | 1.7 | | 61 | | 0.996 |
